# Rationales for the Choice of Metals for Super‐Reduced Biological Metal Centers: Cobalt in Cobalamin Versus Nickel in F_430_

**DOI:** 10.1002/chem.71214

**Published:** 2026-06-03

**Authors:** Radu‐Ioan Onija, Sergiu‐Raul Cosma, Adrian M. V. Brânzanic, Radu Silaghi‐Dumitrescu

**Affiliations:** ^1^ Faculty of Chemistry and Chemical Engineering and INSPIRE Platform Infobionano4health & Biomedical Imaging Babeș‐Bolyai University Cluj‐Napoca Romania; ^2^ National Institute for Research and Development of Isotopic and Molecular Technologies Cluj‐Napoca Romania; ^3^ “Raluca Ripan” Institute for Research in Chemistry Babeș‐Bolyai University Cluj‐Napoca Romania

**Keywords:** cobalamin, cobalt, DFT, F_430_, nickel, super‐reduced

## Abstract

Reported here are DFT calculations on porphyrin, corrin, and corphin complexes with formally metal (I) states of cobalt, rhodium, iron, manganese, or nickel, complemented by QM/MM calculations on enzyme models, and exploring the available spin states, with the goal to understand factors controlling the availably of super‐reduced metal(I) states on such biologically‐relevant systems. In the porphyrin systems, the preferred spin states offer no clear case of metal(I) electronic description—but rather metal(II) coupled with porphyrin anion radicals; this is in line with the fact that porphyrin is not used in biology for mechanisms/reactions entailing super‐reduced metals. The corrin, somewhat less conjugated than the porphyrin, affords a clear‐cut ground‐state S = 0 Co(I) complex, with essentially no delocalization of the extra electron onto the macrocycle. None of the neighbors of cobalt in the third period afford clear super‐reduced ground states with corrin—in line with cobalamin's choice of metal in biology, cobalt. The least conjugated system, the corphin (as seen in F_430_), now affords a metal(I) state even in the case of Ni. Moreover, in fact, only Ni affords a ground state with clear super‐reduced character—in line with F_430_‘s choice of metal in vivo.

## Graphical abstract

DFT calculations on porphyrin, corrin, and corphin complexes of cobalt, rhodium, iron, manganese, or nickel, and QM/MM calculations on enzyme models, explore the factors controlling the availably of super‐reduced metal(I) states. True metal(I) states are seen in corrin only for cobalt, in corphin only for nickel, and not at all in porphyrin.

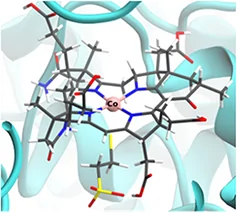

## 1 Introduction

A significant number of enzymes are dependent on cofactors based on tetrapyrrole macrocycles chelating a metal center with the four nitrogen atoms that make up the primary structural characteristic of the ring [1, 2]. Beyond the most common class of such cofactors—the hemes—two other biological metallamacrocyles stand out with an unusual ability to stabilize (and employ mechanistically) super‐reduced metal(I) centers: corrin, found in vitamin B_12_, and corphin, found in coenzyme F_430_. The reaction mechanism of cobalamin within the enzyme methionine synthase (MTR) is characterized by the metal center of cobalt employing a remarkable electron configuration of Co(I), with a fully occupied d_z2_ orbital. This configuration allows the cobalt to act as a super‐nucleophile capable of shuttling methyl cations via cobalt‐carbon bonds. Similarly, F_430_ employs a super‐reduced state for nickel, Ni(I).

A number of studies have previously explored the structural, electronic and metabolic factors that dictate the choice of cobalt in cobalamin and of nickel in F_430_ – and have explored manners in which these metals can be interchanged or replaced synthetically in vitro [3, 4, 5, 6, 7, 8, 9, 10, 11, 12]. Using DFT calculations on metal(1) complexes on small unsusbstituted porphyrin, corrin and corphin models, Jensen and Ryde in particular have found two main factors affecting the electronic structure: (1) the flexibility of the macrocycle (corphin>corrin>porphyrin), (2) size of the inner cavity of the macrocycle (corphin>porphyrin>corrin, favoring higher spin states of Fe in the porphyrin, higher spin states of Ni in the corphin, and lower spin states of Co in the corrin). Implications on the stability of axial Ni‐carbon bonds were discussed, as Ni─CH_3_ reaction intermediates are one of the possible (though not favored [13]) routes in the catalytic cycles of methane/ethane‐forming F_430_‐containing reductases.

Reported here are calculations on tetrapyrrole macrocycle models (porphyrin, corrin, corphin, and related systems), where the central atom is iron, nickel, cobalt, rhodium, or manganese,—and then extension to more realistic models thereof using QM/MM calculations. Reasons for the choice of corrin and corphin in cobalamin and F_430_ as biological centers harboring super‐reduced metal centers are exposed—as well as reasons for the choice of cobalt and nickel, respectively, in those cofactors.

## 2 Results and Discussion

Table 1 shows the DFT‐predicted electronic structures of formally metal(I) corrin adducts, with no axial ligands. For the formally d^6^ Mn(I) corrin models, an expected clear preference for the high‐spin S = 2 state is found—consisting in an S = 3/2 Mn(II) coupled with a one‐electron reduced corrin, cf. Table 1. A similar structure is seen in the S = 1 manganese system, except that now the S = 3/2 Mn(II) system is coupled antiferromagnetically with an S = 1/2 corrin. The S = 0 state features 4 clear electrons on the metal d orbitals; additionally, two more d orbitals have ∼0.5 occupancies. The latter may be interpreted formally as d electrons (in which the S = 0 state would feature a true Mn(I)), or as ligand electrons (hence Mn(III)), or the intermediate situation and similar to the higher‐spin states (hence Mn(II)). In any case, especially considering the spin state preference, the Mn corrin does not appear likely to harbor a true Mn(I) super‐reduced center. For the formally d^7^ Fe(I) corrin models, the two spin states are nearly degenerate, with S = 3/2 slightly favored cf. Table 1. This quartet ground state features an S = 1 d^6^ Fe(II) (cf. Tables S1) coupled to a corrin S = 1/2 radical (cf. Table 1). On the other hand, the doublet state features a clear S = 1/2 d^7^ iron, with the unpaired electron residing in a d_xy_ orbital (cf. Table S1). In the cobalt‐corrin systems (cf. Table 1) the S = 0 state is favored, and features a true d^8^ Co(I) center (cf. Table S1). By contrast, the high‐spin state features a d^7^ electronic structure (S = 1/2 Co(II)) coupled with an unpaired electron on the corrin. The Ni(I) corrin model features a clear S = 0 d^8^ Ni(II) center coupled with a corrin free radical (Table S1). Overall, three of the metals discussed above (manganese, iron and cobalt) feature true super‐reduced states in the low‐spin but not in the higher‐spin states. However, only in the case of cobalt is this low‐spin state also a clear ground state. Nickel(I) only has one possible spin state—which features a corrin‐reduced Ni(II) complex rather than a true super‐reduced center. It may be argued that the sterical constraints of the corrin ring disfavor the lower oxidation states of the metal; this would be more obvious in the high‐spin states, dues to their inherently larger metal radii. In order to further test this hypothesis, a Rh(I)‐corrin model was also examined. Its electronic structures in the S = 0 and S = 1 states, (cf. Tables 1 and S2), are essentially identical to that of its isolectronic counterpart, Co(I)‐corrin, even though Rh is larger than Co (and also larger than Mn, Fe and Ni).

**TABLE 1:** Relative energies and key data on population analyses for corrin adducts.

Metal	Spin	Formal d count	dE (kcal/mol)	Metal charge	Metal spin	Corrin spin	Actual d count
Mn(I)	**0**	**6**	**0**	**0.79**	**0**	**0**	**6**
	1	6	−22.4	0.62	2.54	−0.54	5
	2	6	−35.2	0.87	3.27	0.73	5
Fe(I)	1/2	7	0	0.58	0.83	0.17	7
	3/2	7	−4.5	0.72	2.12	0.88	6
Co(I)	**0**	**8**	**0**	**0.3**	**0**	**0**	**8**
	1	8	6.1	0.54	1.02	0.98	7
Rh(I)	**0**	**8**	**0**	**0.01**	**0**	**0**	**8**
	1	8	28.1	0.34	0.70	1.30	7
Ni(I)	1/2	9	0	0.47	0.02	0.98	8

See Table S1 for details of d orbital occupancies. The lowest spin state is taken as reference for each metal.

The above considerations shed light on the choice of cobalt for cobalamin and specifically for the task of providing a super‐nucleophile center in methyl transferase enzymes using a super‐reduced metal center. A related center, also employing a super‐reduced metal as part of the catalytic cycle, is the F_430_ Ni‐corphin ring. The corphin may be regarded as an even less conjugated version of the corrin ring, compared to porphyrin. Calculations were then performed on metal(I) corphin centers, to see which metals would best accommodate a metal(I) state within a corphin coordination environment. The results are summarized in Tables 2 and S3.

**TABLE 2:** Relative energies and key data on population analyses for corphin adducts.

Metal	Spin	Formal d count	dE(Kcal/mol)	Metal charge	Metal spin	Corphin spin	d count
Mn(I)	**0**	**6**	**0**	**0.5**	**0**	**0**	**6**
	1	6	−41.2	0.8	3.73	−1.73	5
	2	6	−35.6	0.8	3.10	0.90	5
Fe(I)	1/2	7	0	0.72	1.83	−0.83	6
	3/2	7	0.5	0.85	2.42	0.58	6
Co(I)	**0**	**8**	**0**	**0.29**	**0**	**0**	**8**
	1	8	−3.6	0.73	1.17	0.83	7
Rh(I)	**0**	**8**	**0.1**	**0.01**	**0**	**0**	**8**
	1	8	−46.4	0.5	1.06	0.94	7
Ni(I)	**1/2**	**9**	**0**	**0.39**	**1.01**	**−0.01**	**9**

See Table S3 for details of d orbital occupancies. The lowest spin state is taken as reference for each metal.

The manganese corphin adducts exhibit behaviors very similar to the corrin versions discussed above—that is, clear Mn(II) character for the higher‐spin states, and a strongly covalent situation in the much less stable S = 0 state, where any assignment between Mn(I) and Mn(III) can be argued. With iron, a clear total of 6 d electrons can be assigned in the Fe‐corphin system cf. Table S3; an additional electron possibly residing in the β d_xz_ (0.48 occupancy cf. Table S3) may be debated. The two spin states of the Fe complex are, as in the case of the corrin models of Table 1, degenerate energetically. For cobalt, the electronic structures are very similar to those already seen in the corrin system (hence, cobalt(I) in the S = 0 state, and cobalt(II) in the higher‐spin states). However, remarkably, the high‐spin state is now essentially degenerate with the S = 0 – suggesting that cobalt would no longer be able to maintain a clean Co(I) state in a corphin coordination environment. In contrast, as also shown in Tables 2 and S4, nickel is the only case where the corphin coordination environment allows a true Ni(I) S = 1/2 super‐reduced state in both models. Overall, nickel appears as the optimal choice in a corphin environment for reaching low‐valent states.

Previous efforts to compare corrin versus corphin experimentally and to replace cobalt versus nickel [3] have led to the synthesis of a corrin derivative featuring two extra hydroxyl groups. Tables S4 and S5 report DFT calculations on the metal(I) adducts of such corrin derivatives. These results are qualitatively similar to those already discussed above for the corphin case—and, in the case of the nickel system, are in agreement with previously‐reported [3] experimental and DFT data confirming a Ni(I) character. An exception is the Co case, where the di‐hydroxy corrin features a low‐spin ground state similarly to corrin and not to corphin.

The results of calculations on porphyrin metal(I) complexes are shown in Tables 3 and S6. For manganese, the low‐spin state shows a clearer Mn(I) character than in the corrin and corphin derivatives (i.e., 6 d electrons with occupancies of 0.77 or higher, hence a true d^6^ Mn(I) center), but is still distinctly less stable than the higher spin states—and those still feature clear Mn(II) character. In the case of Fe, in line with previously‐discussed data, [14, 15] a Fe(II) character is seen in the high‐spin state, and a more problematic covalently‐mixed state is seen in the low‐spin state. The two spin states are still degenerate, similarly to the corrin and corphin cases. The Ni system shows a clear set of 8 d electrons (cf. Table 1), and an additional 0.5 occupancy in the β d_xz_ orbital. This electronic structure may be interpreted as a mixture between a true S = 1/2 Ni(I) center with its electron delocalized 50% across the porphyrin, vs. an S = 1 Ni(II) coupled to a porphyrin anion radical where the radical is delocalized 50% across the metal.

**TABLE 3:** Relative energies and key data on population analyses for porphyrin adducts.

Metal	Spin	Formal d count	dE (kcal/mol)	Metal charge	Metal spin	Porphyrin spin	Actual d count
Mn(I)	0	6	0.0	0.78	0.00	0.00	6
	1	6	−49.4	0.94	3.57	−1.57	5
	2	6	−43.5	0.90	3.52	0.48	5
Fe(I)	1/2	7	0.0	0.70	2.11	−1.11	7
	3/2	7	0.3	0.72	2.14	0.86	6
Co(I)	0	8	0.0	0.28	0.00	0.00	8
	1	8	−4.5	0.66	1.13	0.87	7
Rh(I)	0	8	0.0	−0.05	0	0	8
	1	8	−28.6	0.48	1.07	0.93	7
Ni(I)	1/2	9	0.0	0.49	1.33	−0.33	9

Overall, none of the porphyrin complexes offer a clear case where a single spin state would be favored with a metal(I) oxidation state without significant delocalization of the unpaired electron(s) across the macrocycle. These findings illustrate why cobalamin‐dependent enzymes and F_430_‐dependent enzymes could not rely on heme macrocycles and instead resort to the corrin and to the corphin systems. The common theme of the latter two macrocycles is the distinctly lower degree of conjugation—and hence distinctly lower ability to delocalize the extra electron upon going from metal(II) to metal(I). This difference implies an incentive for the metal to retain the extra electron in the corrin and corphin systems. To sum up, the highest‐conjugation system, the porphyrin, affords no clear case of metal(I) complex of those examined here. The corrin, somewhat less conjugated, affords a clear‐cut S = 0 Co(I) complex, with essentially no delocalization of the extra electron onto the macrocycle. The least conjugated system, the corphin, now affords a metal(I) state even in the case of Ni.

The above discussions are conducted on simple models of the porphyrin/corrin/corphin macrocycles, where the lateral substituents of the macrocycle are all replaced by hydrogen atoms. One may in principle inquire whether the same conclusions can be qualitatively derived from larger, more realistic models. Tables S7 and S8 show the DFT‐predicted electronic structures of the complete models of cofactor F_430_ with Ni versus Co, which are in qualitative agreement with the conclusions from the smaller models already discussed above. Likewise, for the full cobalamin models data are illustrated in Tables S9–S11.

**FIGURE 1:**
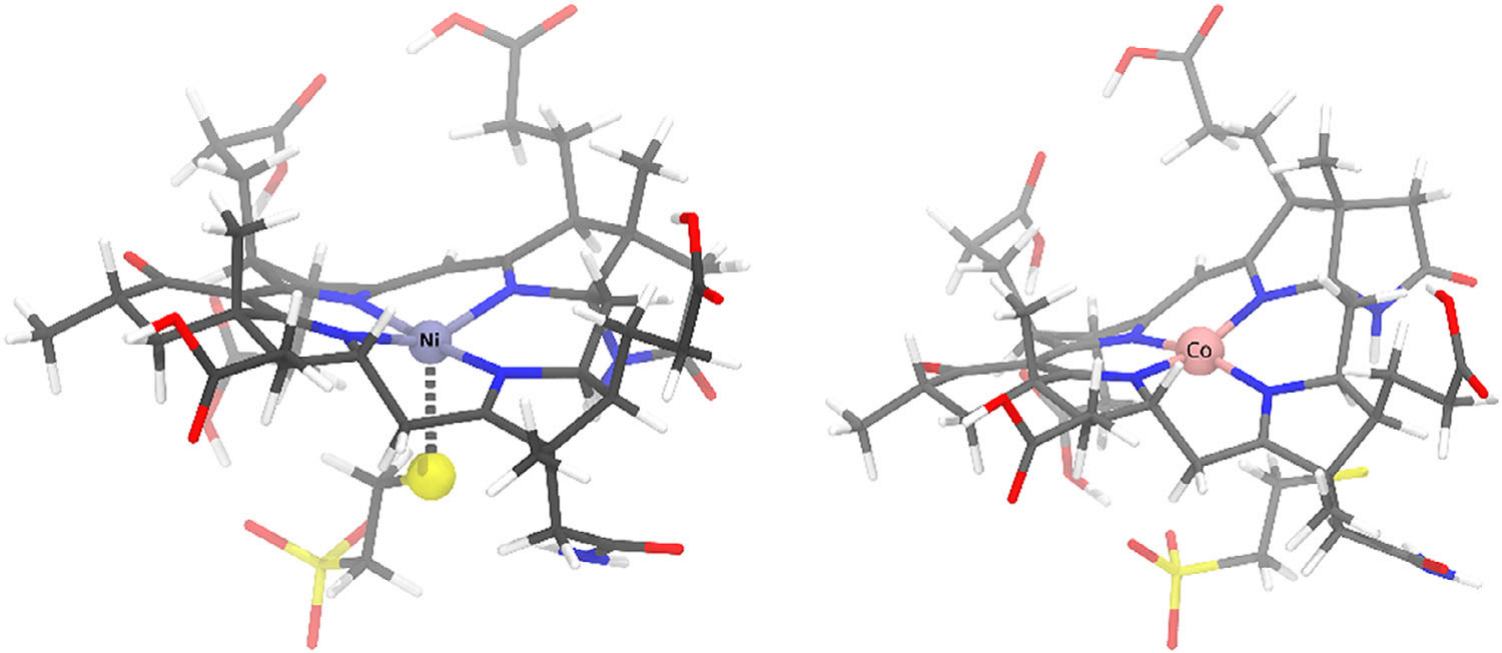
DFT‐optimized full F430 model, complex with Coenzyme M, for Ni versus Co.

A notable point to derive from the full F_430_ and full cobalamin models relates to the ability of Ni, but not of Co, to feature coordination numbers higher than 4 in the super‐reduced state—and to have its electronic structure influenced qualitatively by this extra ligand. Thus, as shown in Figure 1, computations on the F_430_ model also included coenzyme M coordinated to the metal via sulfur, as it is coordinated in the crystal structure of the ethyl‐coenzyme M reductase (ECR) enzyme (from which the model was constructed). This coordination mode was conserved in the Ni model, but not in the Co.

After the DFT characterization of the small and large models, 100 ns of molecular dynamics (MD) and a subsequent QM/MM geometry optimization were performed on the F_430_‐containing ethyl‐coenzyme M reductase (ECR) enzyme comparatively with the native (Ni) and with a Co‐substituted F_430_ cofactor. After the MD calculations the enzyme structures look quite similar (Figure S2), apart from the cofactors, that contain the same structural differences as already illustrated in Figure 1: a 2.45‐Å distance between the Ni and the CoM axial ligand, compared to 3.23 Å in the case of Co. Tables 4 and S11 shows a summary of the key data regarding the Co vs. Ni ECR models. The electronic structures are similar to those seen in the smaller models discussed above.

**TABLE 4:** Relative energies and key data on population analyses for the QM/MM calculations on the Ni and Co‐substituted F_430_ cofactors. For each metal energy values are reported as referenced to the low‐spin state.

Co	Spin	Formal d count	dE (kcal/mol)	Metal spin	Metal charge	Actual d count
Ni	1/2	9	0.0	1.53	0.55	9
Co	0	8	0.0	0.00	0.44	8
	1	8	24.4	2.61	0.62	8

The Supporting Information file also offers an extensive analysis of the macrocycle deformations following Shelnutt's protocols/nomenclature [16]. There appears to be an inverse correlation, expectedly, between the degree of conjugation and the distortions. Lateral substituents and the environment of the protein also provide straightforward routes for altering the distortions of the macrocycle. The identity of the metal is a secondary factor in modulating the distortions. A more extensive systematic analysis of the manners in which a protein environment and a range of lateral and axial substituents may further control these distortions may be warranted.

## 3 Conclusion

To conclude, our DFT, dynamics and QM/MM calculations support the following observations regarding the accessibility of low‐valent states in biological metallomacrocycles:

1. Porphyrin does not accommodate super‐reduced metals.
2. Manganese and iron do not afford clean super‐reduced states in any of the tetrapyrrolic macrocycles.
3. Co and Ni afford super‐reduced states with the right macrocycle. The size of macrocycle and the internal charge (‐2 in porphyrin, ‐1 in corrin/corphin) are important in stabilizing the metal efficiently with as little as possible anionic ligation—so as to favor lower oxidation states. The ability to *not* delocalize the extra electron upon reduction of the divalent metal is also essential; hence the order porphyrin>corrin>corphin holds not only for the extent of the conjugated π/p system, but also for the ability to harbor an anion radical upon reduction of the metal(II) complexes.
4. When a super‐nucleophile/super‐reduced metal is needed for catalysis involving (carbo)cations coordinating to the metal (in effect, reverse coordination, where the metal donates electrons to the ligand), only Co can achieve this task and only with corrin equatorially coordinated.
5. Conversely, for systems that rely on axial coordination from a proper ligand (e.g., sulfur) to the super‐reduced metal super‐reduced metal, only Ni will do, but only with corphin equatorially coordinated as seen in F_430_.

A set of synthetic of metal‐substituted B_12_ analogues is available experimentally from the Krautler group [6, 7, 9, 10, 17, 18, 19] (ferribalamin, zincobyric acid/zincobalamin, nibalamin/nibyric acid, adenosylrhodibalamin) show that the corrin scaffold can host a range of metals geometrically, but that the catalytically meaningful low‐valent (M(I)) chemistry remains essentially Co‐specific. Our calculations rationalize this experimental observation: only Co (and its 4d congener Rh) gives a clear, ground‐state S = 0 d^8^ M(I) center in corrin, while Ni in corrin is consistently described as a corrin‐radical‐reduced Ni(II) complex rather than a true Ni(I). Conversely, the Brenig/Zelder hydroxylated Ni‐corrin construct, [3] which behaves spectroscopically and electrochemically as an F_430_ mimic with metal‐centered reduction, is reproduced here in Tables S4, S5; our DFT data and the experiments agree that the extra OH substituents lower the macrocycle's conjugation toward the corphin regime and recover Ni(I) character. For iron, our finding that no tetrapyrrole studied affords a clean, ground‐state Fe(I) is consistent with the earlier experimental and computational analyses of “super‐reduced” iron in heme environments, [11] where the reduced state was likewise shown to be an electromerically mixed Fe(II)‐porphyrin‐radical situation rather than a localized Fe(I). The previously reported comparative demetallation studies on metalloporphyrins [5, 8] provide indirect support of the same interpretation: porphyrin's strongly conjugated, dianionic cavity binds high‐valent metals tightly, but not super‐reduced congeners.

## Conflicts of Interest

The authors declare no conflicts of interest.

## Supporting information




**Supporting File**: chem71214‐sup‐0001‐SuppMat.docx.

## Data Availability

The data that supports the findings of this study are available in the supplementary material of this article
