# Supplementary material for: Rationales for the Choice of Metals for Super‐Reduced Biological Metal Centers: Cobalt in Cobalamin Versus Nickel in F430
Source: Chemistry. 2026 Jun 3;32(35):e71214. doi: 10.1002/chem.71214 (PMC13587649; doi:10.1002/chem.71214)
Supplement: Supplementary file 1 [file CHEM-32-e71214-s001.docx]

*Supporting Information*

**Rationales for the choice of metals for super-reduced biological metal centers: cobalt in cobalamin vs. nickel in F430**

*Radu-Ioan Onija,^1,2^ Sergiu-Raul Cosma, ^1,3^ Adrian M.V. Brânzanic,^1,3^ Radu Silaghi-Dumitrescu^1,*^*

^1^Faculty of Chemistry and Chemical Engineering and INSPIRE Platform InfoBioNano4Health & Biomedical Imaging, Babeș-Bolyai University, 11 Arany Janos str., 400028 Cluj-Napoca, Romania

^2^ National Institute for Research and Development of Isotopic and Molecular Technologies, Donath 67-103, 400293 Cluj-Napoca, Romania

^3^“Raluca Ripan” Institute for Research in Chemistry, Babeș-Bolyai University, Fântânele 30, 400294 Cluj-Napoca, Romania.

**to whom correspondence should be addressed*

**Materials and Methods**

The initial DFT calculations were performed on small cluster models representing the core tetrapyrrole macrocycles characteristic of the relevant cofactors. These small models included corrin, corphin, porphyrin, and an intermediate di-hydroxy corrin, each coordinated to various metal centers (Mn, Fe, Co, Ni, and Rh) as illustrated in Figure S1. Larger models were constructed to represent the full cofactors as found in their respective enzymes, specifically cofactor F_430_ and vitamin B_12_ (cobalamin), based on experimentally determined structures.[1]

Initial small and large models were constructed using GaussView,[2] which was also used to model metal substitutions. Maestro[3] (Schrödinger suite) was employed to mutate non-standard residues, add hydrogen atoms, and complete missing backbone segments. Visualization of final structures and simulation trajectories was performed using VMD,[4] which was also used to generate the structural diagrams presented in this study.

Parameterization was performed using the antechamber and parmchk2[5, 6] programs from the AMBER[7] suite to generate the initial .frcmod and .mol2 files. DFT calculations and Mulliken analyses were carried out using Gaussian09.[8] The cluster models were optimized at the unrestricted DFT level using the B3LYP[9–11] functional and the def2-SV(P)[12] basis set, with Grimme’s D3 dispersion correction to account for long-range dispersion. Solvation effects in water were modeled using the implicit conductor-like polarizable continuum model (CPCM). Mulliken population analysis was performed to characterize the electron population of the metal centers’d orbitals, particularly in their super-reduced states.

MD simulations were performed using the AMBER suite,[13] employing pmemd.MPI for CPU parallelization and pmemd.cuda for GPU acceleration.

The QM/MM calculation was computed via the use of the ORCA 6.0 program, with the use of the converted amber force field, that was accomplished via the orca_mm module.[14]

**Figure S1.** Models employed in this study. The central metal from the small and large models is substituted with other elements in the same period or group (i.e. Mn, Fe, Co, Ni and Rh).

Molecular Mechanics (MM) calculations were performed using the crystal structure of the ethyl-CoM reductase (ECR) enzyme, employed by anaerobic archaea in the metabolism of ethane within the sea floor,[1] with hydrogen atoms added and missing backbone segments completed. Modified amino acids present in the structure were reverted to their corresponding standard residues. Initial pKa values for all amino acids were calculated at pH 7.4 and protonation states of acidic and basic amino acids were assigned accordingly.

The starting geometry for the QM/MM calculations was taken from the equilibrated structure obtained after MD simulations, ensuring that the system—including solvent and protein—was physically realistic and at an energy minimum. The MD simulations served to resolve any unfavorable contacts in the initial structure, and gradual heating from 0 K to 300 K was performed to prevent instabilities due to sudden increases in kinetic energy.

**Figure S2.** QM regions of the original and Co-substituted F_430_ cofactors. The Ni atom is highlighted in silver, and the Co atom is represented in pink, with the ECR enzyme structure highlighted in cyan.

MM simulations were performed using the AMBER suite, following standard protocols for parameterization and protein minimization. The ff19SB force field was used for the protein and the TIP3P explicit water model[15] for the solvent environment, while the General Amber Force Field (GAFF) was applied to parameterize non-standard residues. The MCPB (Metal Center Parameter Builder)[16] protocol was employed for the metal site parameterization and the MM relaxation protocol recommended by the AMBER developers (cf. <https://ambermd.org/tutorials/basic/tutorial13/index.php>) was further used.

The relaxation protocol consisted of nine steps: (1) minimization of water molecules and ions with the rest of the system being kept restrained; (2) heating the system from 100 K to 298 K at constant volume over 1 ns; (3) equilibration at 298 K and constant pressure to adjust box density; (4) gradual reduction of protein restraints at constant pressure and temperature; (5) minimization with restraints on the protein backbone; (6) further relaxation with the same restraints; (7) reduction of backbone restraint strength from 10 to 1 kcal/mol·Å^2^; (8) further reduction of backbone restraints to 0.1 kcal/mol·Å²; and (9) final unrestrained relaxation for 1 ns at constant pressure.

After the nine-step relaxation process, production MD simulations were performed for 100 ns at constant pressure and temperature, using a periodic box encompassing the protein and solvent. The SHAKE[17] algorithm was used to constrain hydrogen bond lengths, enabling a larger time step of 2 fs.

The force field used in the QM/MM calculations was the same as in the preceding MD simulations, converted to the appropriate format using the orca_mm module in the ORCA suite. In order to provide consistency with the cluster approach, the same DFT methodology was employed on the QM region using the B3LYP/G keyword, to maintain the same functional algorithm used between the two programs. Four QM/MM calculations were performed, substituting Ni with Co in the cofactor at both low-spin and high-spin states.

**Geometry optimization results**

**Table S1.** Occupancies of the metal d orbitals for the corrin adducts. Orbitals formally assigned as occupied are shown in bold. Disputable cases are shown in grey.

| Species | Mn(I ) S=0 | | Mn(I ) S=1 | | Mn(I ) S=2 | | Fe(I) S=1/2 | | Fe(I) S=3/2 | | Co(I) S=0 | | Co(I) S=1 | | Co(I) S=2 | | Ni(I) S=1/2 | |
| --- | --- | --- | --- | --- | --- | --- | --- | --- | --- | --- | --- | --- | --- | --- | --- | --- | --- | --- |
| Orbital | α | β | α | β | α | β | α | β | α | β | α | β | α | β | α | β | α | β |
| d_xy_ | 0.26 | 0.26 | **0.88** | 0.26 | **0.92** | 0.05 | **0.89** | 0.12 | **0.93** | 0.17 | **0.87** | **0.87** | **0.93** | **0.88** | **0.96** | 0.40 | **0.93** | **0.93** |
| d_xz_ | **0.83** | **0.83** | **0.91** | 0.35 | **0.92** | **0.70** | **0.86** | **0.83** | **0.94** | **0.90** | **0.85** | **0.85** | **0.94** | **0.92** | **0.97** | **0.94** | **0.95** | **0.95** |
| d_yz_ | **0.49** | **0.49** | **0.86** | 0.03 | **0.99** | 0.05 | **0.63** | **0.78** | **0.99** | 0.05 | **0.81** | **0.81** | **0.99** | 0.10 | **0.99** | 0.06 | **0.98** | **0.96** |
| d_x2-y2_ | **0.90** | **0.90** | **0.95** | **0.68** | **0.97** | 0.05 | **0.96** | **0.95** | **0.97** | **0.82** | **0.96** | **0.96** | **0.97** | **0.97** | **0.98** | **0.59** | **0.97** | **0.97** |
| d_z2_ | 0.27 | 0.27 | 0.31 | 0.25 | 0.39 | 0.29 | 0.29 | 0.27 | 0.35 | 0.28 | 0.30 | 0.30 | 0.38 | 0.32 | **1.02** | 0.30 | 0.39 | 0.39 |

**Table S2.** Occupancies for the S=0 rhodium species. Orbitals formally assigned as occupied are shown in bold. Disputable cases are shown in grey.

| Species | Corrin | | Di hydroxy corrin S=0 | | Di hydroxy corrin S=1 | | Corphin S=0 | | Corphin S=1 | | Porphyrin S=0 | | Porphyrin S=1 | |
| --- | --- | --- | --- | --- | --- | --- | --- | --- | --- | --- | --- | --- | --- | --- |
| Orbital | α | β | α | β | α | β | α | β | α | β | α | β | α | β |
| d_xy_ | **0.88** | **0.88** | **0.88** | **0.88** | **0.93** | 0.25 | **0.83** | **0.83** | **0.94** | 0.14 | **0.89** | **0.89** | **0.94** | **0.10** |
| d_xz_ | **0.88** | **0.88** | **0.88** | **0.88** | **0.98** | **0.88** | **0.89** | **0.89** | **0.98** | **0.93** | **0.89** | **0.89** | **1.00** | **0.95** |
| d_yz_ | **0.86** | **0.86** | **0.88** | **0.88** | **0.96** | **0.89** | 0.40 | 0.40 | **0.97** | **0.94** | **0.89** | **0.89** | **0.97** | **0.97** |
| d_x2-y2_ | **0.98** | **0.98** | **0.92** | **0.92** | **0.89** | **0.88** | 0.38 | 0.38 | **0.89** | **0.89** | 0.46 | 0.46 | 0.39 | 0.37 |
| d_z2_ | 0.36 | 0.36 | 0.41 | 0.41 | **0.50** | **0.48** | **0.90** | **0.90** | **0.50** | **0.47** | **0.86** | **0.86** | **0.99** | **0.99** |

**Table S3.** Occupancies of the metal d orbitals for the corphin adducts. Orbitals formally assigned as occupied are shown in bold. Disputable cases are shown in grey.

| Species | Mn(I ) S=0 | | Mn(I ) S=1 | | Mn(I ) S=2 | | Fe(I) S=1/2 | | Fe(I) S=3/2 | | Co(I) S=0 | | Co(I) S=1 | | Co(I) S=2 | | Ni(I) S=1/2 | |
| --- | --- | --- | --- | --- | --- | --- | --- | --- | --- | --- | --- | --- | --- | --- | --- | --- | --- | --- |
| Orbital | α | β | α | β | α | β | α | β | α | β | α | β | α | β | α | β | α | β |
| d_xy_ | **0.76** | **0.76** | **0.93** | 0.05 | **0.94** | 0.04 | **0.93** | 0.05 | **0.95** | **0.40** | **0.87** | **0.87** | **0.95** | **0.91** | **0.97** | **0.74** | **0.92** | **0.88** |
| d_xz_ | **0.48** | **0.48** | **0.96** | 0.08 | **0.98** | 0.03 | **0.96** | **0.48** | **0.98** | 0.29 | **0.82** | **0.82** | **0.98** | 0.31 | **0.99** | 0.21 | **0.95** | **0.84** |
| d_yz_ | 0.33 | 0.33 | **0.96** | 0.10 | **0.96** | 0.06 | **0.94** | **0.71** | **0.98** | 0.36 | **0.91** | **0.91** | **0.99** | 0.12 | **0.99** | 0.15 | **0.97** | **0.93** |
| d_x2-y2_ | **0.84** | **0.84** | **0.87** | **0.72** | **0.86** | **0.74** | **0.90** | **0.84** | **0.99** | 0.16 | **0.63** | **0.63** | **0.98** | **0.74** | **0.99** | **0.80** | **0.98** | **0.72** |
| d_z2_ | 0.34 | 0.34 | 0.39 | 0.32 | **0.40** | 0.33 | 0.37 | 0.32 | **0.99** | 0.18 | 0.58 | 0.58 | **0.99** | 0.38 | **1.00** | 0.32 | **0.99** | 0.39 |

**Table S4.** Relative energies and key data on population analyses for di-hydroxy corrin adducts

| Metal | Spin | Formal d count | dE (kcal/mol) | Metal charge | Metal spin | Corrin spin | Actual d count |
| --- | --- | --- | --- | --- | --- | --- | --- |
| Mn(I) | 0 | 6 | 0 | 0.49 | 0 | 0 | 6 |
|  | 1 | 6 | -36.7 | 0.81 | 3.19 | -1.19 | 5 |
| Fe(I) | 1/2 | 7 | 0 | 0.54 | 1.83 | -0.83 | 6 |
|  | 3/2 | 7 | 9.9 | 0.57 | 2 | 1 | 6 |
| Co(I) | 0 | 8 | 0 | 0.28 | 0 | 0 | 8 |
|  | 1 | 8 | 11.9 | 0.65 | 1.15 | 0.85 | 7 |
| Rh(I) | 0 | 8 | 0.0 | 0 | 0 | 0 | 8 |
|  | 1 | 8 | 46.4 | 0.40 | 1 | 1 | 7 |
| Ni(I) | 1/2 | 9 | 0 | 0.34 | 0.95 | 0.05 | 9 |

**Table S5**. Occupancies for the metal d orbitals for the di hydroxy corrin adducts. Orbitals formally assigned as occupied are shown in bold. Disputable cases are shown in grey.

| Species | Mn(I) S=0 | | Mn(I) S=1 | | Fe(I) S=1/2 | | Fe(I) S=3/2 | | Co(I) S=0 | | Co(I) S=1 | | Co(I) S=2 | | Ni(I) S=1/2 | |
| --- | --- | --- | --- | --- | --- | --- | --- | --- | --- | --- | --- | --- | --- | --- | --- | --- |
| Orbital | α | β | α | β | α | β | α | β | α | β | α | β | α | β | α | β |
| d_xy_ | **0.74** | **0.74** | **0.92** | 0.08 | **0.91** | **0.68** | **0.92** | **0.83** | **0.87** | **0.87** | **0.94** | 0.14 | **0.97** | 0.52 | **0.92** | **0.88** |
| d_xz_ | **0.59** | **0.59** | **0.96** | 0.29 | **0.96** | 0.19 | **0.98** | 0.04 | **0.84** | **0.84** | **0.98** | **0.85** | **0.99** | 0.35 | **0.95** | **0.86** |
| d_yz_ | 0.25 | 0.25 | **0.95** | 0.37 | **0.94** | 0.26 | **0.96** | 0.10 | **0.84** | **0.84** | **0.96** | **0.93** | **0.99** | 0.33 | **0.96** | **0.85** |
| d_x2-y2_ | **0.56** | **0.56** | **0.91** | 0.20 | **0.92** | **0.90** | **0.89** | **0.87** | **0.92** | **0.92** | **0.89** | **0.88** | **0.98** | **0.82** | **0.96** | **0.94** |
| d_z2_ | **0.61** | **0.61** | **0.43** | 0.25 | 0.38 | 0.31 | **0.43** | 0.35 | 0.33 | 0.33 | 0.44 | 0.40 | **1.00** | 0.24 | **0.97** | 0.24 |

**Table S6.** Occupancies for the metal d orbitals for the porphyrin adducts. Orbitals formally assigned as occupied are shown in bold. Disputable cases are shown in grey.

| Species | Mn(I ) S=0 | | Mn(I ) S=1 | | Mn(I ) S=2 | | Fe(I) S=1/2 | | Fe(I) S=3/2 | | Co(I) S=0 | | Co(I) S=1 | | Co(I) S=2 | | Ni(I) S=1/2 | |
| --- | --- | --- | --- | --- | --- | --- | --- | --- | --- | --- | --- | --- | --- | --- | --- | --- | --- | --- |
| Orbital | α | β | α | β | α | β | α | β | α | β | α | β | α | β | α | β | α | β |
| d_xy_ | 0.04 | 0.04 | **0.95** | 0.04 | **0.94** | 0.04 | **0.94** | 0.03 | **0.94** | 0.04 | **0.87** | **0.87** | **0.95** | 0.03 | **0.97** | 0.37 | **0.94** | **0.91** |
| d_xz_ | **0.77** | **0.77** | **0.99** | 0.27 | **0.99** | 0.03 | **0.98** | 0.51 | **0.99** | 0.05 | **0.85** | **0.85** | **0.99** | **0.96** | **0.99** | **0.98** | **0.98** | **0.96** |
| d_yz_ | **0.77** | **0.77** | **0.99** | 0.27 | **0.98** | **0.61** | **0.98** | 0.51 | **0.97** | **0.90** | **0.85** | **0.85** | **0.98** | **0.97** | **1.00** | 0.03 | **0.98** | **0.50** |
| d_x2-y2_ | 0.28 | 0.28 | **0.79** | 0.10 | 0.37 | 0.25 | **0.60** | 0.56 | 0.35 | 0.28 | 0.28 | 0.28 | 0.31 | 0.26 | **0.98** | **0.62** | **1.01** | 0.19 |
| d_z2_ | **0.89** | **0.89** | **0.61** | 0.18 | **0.98** | 0.01 | **0.67** | **0.63** | **0.93** | **0.92** | **0.92** | **0.92** | **0.98** | **0.97** | **1.01** | 0.22 | **0.98** | **0.97** |

**Table S7.** Relative energies and key data on population analyses for F_430_ adducts.

| Metal | Spin | Formal d count | dE (kcal/mol) | metal charge | metal spin | corphin spin | actual d count |
| --- | --- | --- | --- | --- | --- | --- | --- |
| Co | 0 | 8 | 0 | 0.32 | 0 | 0 | 8 |
|  | 1 | 8 | -9.4 | 0.66 | 1.13 | 0.87 | 7 |
| Ni | 1/2 | 9 | 0 | 0.43 | 0.7 | 0.3 | 9 |

**Table S8**. Occupancies for the metal d orbitals for the large F_430_ model adducts. Orbitals formally assigned as occupied are shown in bold.

| Species | Co(I) 0 | | Co(I) 1 | | Ni(I) 1/2 | |
| --- | --- | --- | --- | --- | --- | --- |
| orbital | α | β | α | β | α | β |
| d_xy_ | **0.88** | **0.88** | **0.95** | 0.09 | **0.91** | **0.91** |
| d_xz_ | **0.89** | **0.89** | **0.95** | **0.95** | **0.95** | **0.91** |
| d_yz_ | **0.78** | **0.78** | **0.98** | **0.90** | **0.83** | **0.89** |
| d_x2-y2_ | 0.32 | 0.32 | 0.40 | 0.35 | **0.96** | 0.28 |
| d_z2_ | **0.90** | **0.90** | **0.92** | **0.91** | **0.96** | **0.91** |

**Table S9.** Relative energies and key data on population analyses for the full cobalamin adducts, base-on vs. base-off.

| Species | base | Spin | formal d count | dE (kcal/mol) | metal charge | metal spin | corrin spin | actual d count |
| --- | --- | --- | --- | --- | --- | --- | --- | --- |
| Mn(I) | off | 0 | 6 | 0 | 0.78 | 0 | 0 | 6 |
|  | **on** | **0** | **6** | **0** | **0.56** | **0** | **0** | **6** |
|  | on | 1 | 6 | -0.74 | 0.64 | 1.37 | 1.63 | 5 |
| Fe(I) | **off** | **1/2** | **7** | **0** | **0.58** | **0.93** | **0.07** | **-** |
|  | off | 3/2 | 7 | -5.62 | 0.71 | 2.12 | 0.88 | 6 |
|  | **on** | **1/2** | **7** | **0** | **0.49** | **0.05** | **0** | **6** |
|  | on | 3/2 | 7 | -15.2 | 0.56 | 2.06 | 0.94 | 6 |
| Co(I) | **off** | **0** | **8** | **0** | **0.32** | **0** | **0** | **8** |
|  | off | 1 | 8 | -8.03 | 0.52 | 1.03 | 0.97 | 7 |
|  | on | 0 | 0 | 0 | 0.24 | 0 | 0 | 8 |
| Ni(I) | **off** | **1/2** | **9** | **0** | **0.46** | **0.02** | **0.98** | **8** |
|  | **on** | **1/2** | **0** | **0** | **0.48** | **0.04** | **0.96** | **9** |

**Table S10**. Occupancies for the metal d orbitals for the large base-off cobalamin model adducts. Orbitals formally assigned as occupied are shown in bold. Disputable cases are shown in grey.

| Species | Co(I) 0 | | Co(I) 1 | | Fe(I) 1/2 | | Fe(I) 3/2 | | Mn(I) 0 | | Mn(I) 1 | | Ni(I) 1/2 | |
| --- | --- | --- | --- | --- | --- | --- | --- | --- | --- | --- | --- | --- | --- | --- |
| orbital | α | β | α | β | α | β | α | β | α | β | α | β | α | β |
| d_xy_ | **0.84** | **0.84** | **0.93** | 0.58 | **0.89** | 0.44 | **0.93** | 0.52 | 0.42 | 0.42 | 0.46 | 0.46 | **0.94** | **0.92** |
| d_xz_ | **0.80** | **0.80** | **0.88** | **0.70** | **0.86** | 0.42 | **0.92** | 0.37 | 0.48 | 0.48 | 0.43 | 0.43 | **0.88** | **0.87** |
| d_yz_ | **0.81** | **0.81** | **0.96** | **0.66** | **0.61** | **0.76** | **0.92** | 0.18 | **0.62** | **0.62** | 0.64 | 0.64 | **0.97** | **0.94** |
| d_x2-y2_ | 0.59 | 0.59 | **0.62** | 0.55 | 0.60 | 0.58 | 0.66 | 0.56 | **0.57** | **0.57** | 0.53 | 0.53 | 0.64 | 0.65 |
| d_z2_ | **0.75** | **0.75** | **0.82** | **0.70** | **0.70** | **0.69** | **0.74** | 0.57 | **0.69** | **0.69** | 0.71 | 0.71 | **0.79** | **0.82** |

**Table S11.** Summary information regarding the QM/MM calculations for the original and Ni-substituted F_430_ cofactor within the ECR enzyme

| Metal | Spin | Formal d count | QM Energy (Hartree) | MM Energy (Hartree) | Total Energy | dE (kcal/mol) | Metal Spin | Metal Charge | Actual d count |
| --- | --- | --- | --- | --- | --- | --- | --- | --- | --- |
| Ni | 1/2 | 9 | -5623.2 | -142.79 | -5765.97 | 0.00 | 1.53 | 0.55 | 9 |
| Co | 0 | 8 | -5497.9 | -952.78 | -6450.71 | 0.00 | 0.00 | 0.43 | 8 |
|  | 1 | 8 | -5496.0 | -952.78 | -6449.75 | 24.3 | 2.61 | 0.62 | 8 |

**Macrocycle distortions**

*General comments*

Direct structural support for the role of conjugation in super-reduction capacity is obtained from a normal-coordinate structural decomposition of all DFT-optimised geometries, following the framework of Jentzen, Song and Shelnutt.[18] The 24-atom inner ring of porphyrin and corphin (4 N + 8 Cα + 4 Cmeso + 8 Cβ) and the 23-atom inner ring of corrin (the latter with a direct Cα–Cα closure replacing one of the meso bridges) were identified by graph topology and fitted to a least-squares mean plane. The per-atom displacements perpendicular to this plane (the out-of-plane Δz) and within it (the in-plane Δx, Δy) were projected onto the lowest-order symmetry-adapted minimal basis of the planar D4h-symmetric macrocycle: saddled, ruffled, domed, waved and propellered for the out-of-plane component, and breathing, N-stretched, meso-stretched, rotated and translated for the in-plane component. This analytic minimal basis was chosen for methodological uniformity across all nine datasets analyzed (porphyrin small models, two Rh-only macrocycle-class probes - bacteriochlorin and an amide-substituted porphyrinoid — four corrin variants, and two corphin coordination states The dataset additionally includes a 4d-metal (rhodium) extension spanning every macrocycle class in this work (three Rh-porphine spin states, four Rh-corrin and three Rh-di-OH-corrin small models, a Rh-substituted F430 cofactor, plus three Rh-bacteriochlorin and three amide-substituted-porphyrinoid (1HBM_Rh) models that test how peripheral saturation and peripheral amide substitution modulate macrocyclic shape), which extends the structural decomposition from a purely 3d-metal series to a 4d/3d comparison. The maximum out-of-plane atomic displacement increases steeply with decreasing conjugation: porphyrin (below 3 mÅ for all thirteen metal/spin combinations) ≪ corrin (up to 0.68 Å in the small di-hydroxy models, up to 0.86 Å in the decoordinated full cobalamin) ≪ corphin (up to 1.18 Å in the full F430 models), providing a direct geometric counterpart to the electronic argument given above. Within each corrin model size — bare, di-hydroxy and decoordinated full cobalamin — the three high-distortion outliers identified by the decomposition (Co(I)/S=2, Mn(I)/S=0 and Ni(I)/S=3/2) coincide with the three spin states for which the orbital analyses do not afford a clean super-reduced character; the Co(I)/S=2 and Ni(I)/S=3/2 cases share a saddled + domed signature with a sign-flip in the propellering mode that is unique to them and additionally displace the metal 0.4–0.5 Å out of the macrocyclic plane. The peripheral side chains of the cobalamin cofactor in the decoordinated state enforce a saddled distortion of the corrin ring: every metal/spin combination of the full cobalamin model adopts a saddled conformation as its dominant out-of-plane mode (Dop of 1.31–1.92 Å), distinct from the mode-spread small-model conformations. Adding the natural axial coordination of the cobalamin cofactor — water above the corrin and dimethylbenzimidazole nitrogen below — reverses this saddling: the total out-of-plane distortion drops to 0.94–1.22 Å and the conformation shifts to a mixed ruffled + propellered character. Peripheral substitution acts on the porphyrin macrocycle in a manner directly analogous to cobalamin's side chains on corrin: a Rh-porphyrinoid with two fused amide pendants (1HBM_Rh, three spin states) is strongly saddled at d_sad = +1.14–1.38 Å across every spin state, while bare Rh-porphine in the same set is essentially planar — peripheral chemistry therefore enforces saddling on both intrinsically-planar (porphyrin) and intrinsically-non-D4h (corrin) host macrocycles, while axial coordination on cobalamin attenuates the saddled distortion that the side chains have already imposed. The asymmetric axial coordination pulls the metal out of the macrocyclic plane on average +0.20 Å (toward the H_2_O above); the Ni(I)/S=3/2 anomaly persists in the decoordinated full cobalamin, where it remains the only complex with a saddled + domed conformation and an unusually large metal-out-of-plane displacement (0.47 Å). Within the corphin / F430 series, the native S=½ Ni(I) complex is the least distorted (max |Δz| = 0.63 Å, Dop = 1.58 Å, conformation saddled + domed + waved), with nickel sitting 0.16 Å above the mean plane — consistent with axial coenzyme-M coordination in the enzyme; both nickel complexes are saddling-amenable with negligible ruffling (|d_ruf| ≤ 24 % of |d_sad|), while most other metal/spin combinations in the corphin set show mixed ruffled + saddled character with substantially larger total distortion — a structural expression of corphin's biological selectivity for nickel. The in-plane analysis reinforces this selectivity: among the corphin set, F430_Ni2 and F430_Ni4 have the smallest in-plane distortions (Dip = 0.36–0.40 Å) while F430_Co5 has the largest (0.78 Å), so cobalt substitution drives both larger out-of-plane AND larger in-plane deformation. The same analysis on the QM/MM-relaxed F430 cofactor in ethyl-coenzyme M reductase (Tables 4 and S11) confirms a 2.44 Å Ni–S axial bond length for the native Ni(I)/S=½ state versus 2.94 Å for the cobalt-substituted Co(I)/S=0 state. The high-spin Co(I)/S=1 substitution recovers close axial coordination at 2.45 Å but at the cost of pushing the metal 0.62 Å out of the macrocyclic plane (the largest M-plane displacement of the four QM/MM cases) — native Ni uniquely reaches the geometric optimum of close axial bonding AND moderate out-of-plane displacement, an optimum that cobalt cannot match in either spin state. The protein environment increases the macrocyclic distortion (Dop of 1.85 Å in the enzyme versus 1.58 Å for the free Ni cofactor) and adds a ruffling component absent in the gas-phase model. Finally, the same analysis identifies a textbook B1g (N-stretched) Jahn–Teller distortion of +0.076 Å in the S=½ nickel–porphyrin complex, the only canonical 3d porphyrin showing such a distortion (Rh5_Pro shows a comparable +0.074 Å N-stretching but in a forced-quintet 4d state), geometrically confirming the 50/50 admixture of S=½ Ni(I) and S=1 Ni(II) + porphyrin-radical character anticipated electronically (Tables 3 and S6).

*Methodological details of macrocycle deformation*

All DFT-optimized geometries were analyzed by a normal-coordinate structural decomposition (NSD) of the macrocyclic inner ring, following Jentzen, Song & Shelnutt.[18] The macrocyclic distortion is decomposed into a fixed minimal basis of low-frequency normal modes derived from the planar D4h-symmetric reference geometry of the macrocycle. The protocol is available publicly as software registered at <https://github.com/branzanic/sofia>. Sofia, the open-source tool used for NSD analysis in this work (<https://github.com/branzanic/sofia)>, was developed collaboratively by [AMVB] and Anthropic's Claude (Opus 4.7) during 2026. The NSD computations performed for the structures in this paper are deterministic linear-algebra projections that depend only on the input geometries; Claude's contribution was to the design and implementation of the software, not to perform the per-molecule analysis.

For each Gaussian opt log the last 'Standard orientation' geometry was read; for ORCA QM/MM jobs, the .QMRegion.xyz file of the QM region was read. The macrocyclic inner ring was identified by graph walking from the metal center through nitrogen-bonded pyrrole 5-rings, automatically rejecting axial ligands, side chains, and (for cobalamin) the dimethylbenzimidazole nucleotide loop. Atom classes (N, Cα, Cmeso, Cβ) were assigned by topological position; the macrocycle was rotated into a canonical D4h-aligned orientation by averaging N positions onto the symmetry-axis directions before projection.

The macrocyclic mean plane was fitted by singular-value decomposition; the molecule was rotated so that the plane normal coincides with the global +z. Out-of-plane (OOP) distortions were defined as the signed atomic z-displacements from the fitted plane. The analytic D4h-pattern minimal basis was constructed from the lowest-order polynomial generators of each irreducible representation using only x,y of the 24 inner-ring atoms: B2u saddling (xy on Cβ pairs), B1u ruffling (xy on Cmeso and Cα pairs), A2u doming (uniform z), Eg waving (x and y), A1u propellering (xy(x²−y²) on Cβ atoms). In-plane (IP) distortions were decomposed into A1g breathing (≈ 0 by orbit-symmetric reference choice), B1g N-stretching, B2g meso-stretching, A2g rotation and Eu translation.

Total distortion magnitudes are the Euclidean norm of each component: D_op = [Σ_i (Δzi)²]^½ and D_ip = [Σ_i (Δxi)² + (Δyi)²]^½, both computed on the 24-atom (porphyrin-class) or 23-atom (corrin) macrocyclic inner ring only. The minimal-basis modes, basis-construction variants and conformer-label syntax used throughout this Section are defined below in Tables S12-S14.

**Table S12.** Macrocyclic distortion modes used as the NSD minimal basis. Out-of-plane (OOP) modes describe atomic z-displacements from the macrocyclic mean plane; in-plane (IP) modes describe (x,y) displacements within that plane. Mode amplitudes are reported in Å. Abbreviations used in subsequent tables: 'sad' = saddled, 'ruf' = ruffled, 'dom' = domed, 'wav' = waved, 'pro' = propellered, 'bre' = breathing, 'N-str' = N-stretched, 'm-str' = meso-stretched, 'rot' = rotated, 'trn' = translated. The minimal-basis comprises ten low-frequency normal modes of the planar D4h-symmetric reference. The labels are inherited from the porphyrin NSD literature.[18] The OOP modes are listed in increasing irrep label order (B2u, B1u, A2u, Eg, A1u). The IP modes are listed by (gerade then ungerade).

| **abbr.** | **name** | **irrep (D4h)** | **type** | **description** |
| --- | --- | --- | --- | --- |
| sad | saddled | B2u | OOP | alternating up/down displacement of the four pyrroles |
| ruf | ruffled | B1u | OOP | alternating up/down displacement of the four meso carbons |
| dom | domed | A2u | OOP | uniform doming/cupping of the inner ring |
| wav(x), wav(y) | waved | Eg (×2) | OOP | wave-like z-displacement pattern (two degenerate components) |
| pro | propellered | A1u | OOP | rotational z-displacement of the eight Cβ atoms |
| bre | breathing | A1g | IP | uniform expansion/contraction of all classes (≈ 0 by ref choice) |
| N-str | N-stretched | B1g | IP | trans-pyrrole pair stretches; alternating N-N elongation |
| m-str | meso-stretched | B2g | IP | alternating Cmeso-Cmeso elongation |
| rot | rotated | A2g | IP | rotation of all four classes about the metal centre |
| trn(x), trn(y) | translated | Eu (×2) | IP | uniform translation of macrocycle vs metal |

Four basis variants were considered for the NSD minimal basis. The choice between them affects only the assignment of signed amplitudes to mode labels; the per-irrep magnitudes |D^Γ| and the geometric quantities (D_op, D_ip, max|dz|, M-plane offset, M-N distances, mean-plane fit) are basis-independent and identical across all four variants. The reported tables and figures throughout this Section use Variant (i).

Table S13. Summary of the four NSD-basis variants considered in this work, the macrocycles to which each was applied, and the eigenvector source. Variant (i) is the analytic D4h-pattern minimal basis used throughout; Variant (ii) is the Gaussian-freq variant for cross-validation.

| **variant** | **eigenvector source** | **applied to** | **role in this work** |
| --- | --- | --- | --- |
| (i) analytic D4h | low-order polynomial generators (xy, xy(x²−y²), uniform z, etc.) | all macrocycles | MAIN BASIS — used throughout |
| (ii) Gaussian freq | Gaussian-computed normal modes from an opt+freq job | Co1corrinOH only (corrin) | cross-validation; nsd_corrin_freq.py |

Why Variant (i) was selected for the main reporting: (a) Methodologically uniform: the same construction applies to porphyrin, corrin, corphin, and to ECR QM/MM geometries, which is essential for cross-dataset comparison. (b) No DFT freq cost: the analytic basis can be built from any optimised geometry without an additional freq job. (c) Closed-form: the basis is unambiguous and reproducible, with no dependence on functional, basis set, or solver settings.

Basis-independence of the structural conclusions. Within each 1-D irrep (B2u sad, B1u ruf, A2u dom, A1u pro, A1g bre, B1g N-str, B2g m-str, A2g rot) the mode is unique up to sign, so the absolute amplitude is fixed. For the 2-D irreps Eg (wav) and Eu (trn), the magnitude |wav| = √(d_wav_x² + d_wav_y²) and |trn| = √(d_trn_x² + d_trn_y²) are basis-rotation-invariant. Conformer labels and structural conclusions are therefore independent of the basis-construction choice.

Every NSD table reports two conformer labels: an out-of-plane (OOP) label and an in-plane (IP) label. Each label is a string of mode abbreviations joined by '+' signs.

Construction rules. (i) Largest-amplitude mode first. (ii) Any other mode whose amplitude is at least 30 % of the largest in absolute value is appended in decreasing order. (iii) If the largest amplitude is below the planarity threshold (0.05 Å, ≈ X-ray positional uncertainty), the label is 'essentially planar' (OOP) or 'essentially symmetric' (IP); sub-threshold residuals are not annotated since they are at or below the noise level of the underlying geometry. (iv) Mode abbreviations are as in Table S14.

**Table S14.** Conformer-label syntax used throughout this Section. The label is a compact textual fingerprint of the macrocyclic distortion that highlights the dominant mode and any secondary contribution above 30 % of the dominant amplitude.

| **conformer label** | **interpretation** | **example** |
| --- | --- | --- |
| essentially planar | all OOP amplitudes < 0.05 Å (X-ray noise) | Co1_Pro, Ni2_Pro, Rh1_Pro: bare metalloporphine |
| essentially symmetric | all IP amplitudes < 0.05 Å | porphyrin set (every entry except Ni2_Pro N-str) |
| sad | single dominant mode | F430_Co1: pure saddling at sad = −2.43 Å |
| ruf + sad | two modes; secondary ≥ 30 % of dominant | F430_Mn1: ruf = −1.55 Å, sad = +0.75 Å (48 % of \|ruf\|) |
| sad + ruf + dom + wav | four modes; complex distortion | F430_Mn5: corphin under high-spin Mn(II) |
| pro + ruf + sad + wav | corrin-class four-mode mix | Rh1corrinOH: di-OH corrin |
| m-str + trn + N-str | in-plane combination | F430_Co1 IP fingerprint |

All twelve metal-porphine complexes are essentially planar by every macroscopic metric (max|dz| ≤ 2 mÅ, D_op ≤ 6 mÅ, D_ip ≤ 82 mÅ). The OOP minimal-basis amplitudes are below 0.005 Å for every mode and every complex. The only IP mode above the 0.05 Å threshold is N-str = +0.076 Å for Ni2_Pro (the canonical Ni(I)/S=½ B1g Jahn–Teller distortion; Table 3 discusses this state). Bare metal-porphine is intrinsically planar regardless of metal (3d Co/Fe/Mn/Ni or 4d Rh) or spin state. Class radii rN = 1.98–2.07 Å, with Rh slightly elongated relative to the low-spin 3d analogues (Rh1: 2.04 Å, Rh3: 2.07 Å vs Co1: 1.98 Å, Mn1: 2.01 Å, Fe2: 2.01 Å).

**Table S15.** NSD amplitudes (Å, signed) and total distortion magnitudes for twelve metal-porphine complexes (Mn/Fe/Co/Ni at S=0/½/1/3/2/2 as applicable, Rh(I) at S=0 and S=1; matches MS Table 3). Headers: file = log file stem; M = metal; S = electronic spin; max|dz| = maximum absolute z-displacement of any inner-ring atom from the mean plane; D_op = Euclidean norm of OOP displacements; D_ip = Euclidean norm of IP displacements; M-plane = signed M-to-mean-plane offset; M-N = mean M-to-N distance; sad/ruf/dom/|wav| = signed amplitudes / magnitudes of the saddled/ruffled/domed/waved minimal-basis modes (Å); OOP/IP conformer = conformer-label fingerprints.

| **file** | **M** | **S** | **max\|dz\|** | **D_op** | **D_ip** | **M-plane** | **M-N** | **sad** | **ruf** | **dom** | **\|wav\|** | **OOP conformer** | **IP conformer** |
| --- | --- | --- | --- | --- | --- | --- | --- | --- | --- | --- | --- | --- | --- |
| Co1_Pro | Co | S=0 | 0.002 | 0.004 | 0.001 | -0.00 | 1.979 | -0.000 | +0.004 | +0.000 | 0.000 | planar | symm |
| Co3_Pro | Co | S=1 | 0.002 | 0.005 | 0.043 | +0.00 | 2.009 | +0.000 | -0.005 | +0.000 | 0.000 | planar | symm |
| Co5_Pro | Co | S=2 | 0.001 | 0.002 | 0.036 | +0.00 | 2.069 | +0.000 | -0.002 | -0.000 | 0.001 | planar | symm |
| Fe2_Pro | Fe | S=1/2 | 0.001 | 0.004 | 0.001 | -0.00 | 2.013 | -0.000 | +0.004 | +0.000 | 0.000 | planar | symm |
| Fe4_Pro | Fe | S=3/2 | 0.002 | 0.004 | 0.034 | -0.00 | 2.023 | -0.000 | +0.004 | +0.000 | 0.000 | planar | symm |
| Mn1_Pro | Mn | S=0 | 0.002 | 0.004 | 0.002 | -0.00 | 2.012 | -0.001 | +0.004 | +0.000 | 0.001 | planar | symm |
| Mn3_Pro | Mn | S=1 | 0.002 | 0.004 | 0.001 | -0.00 | 2.016 | +0.000 | -0.004 | +0.000 | 0.000 | planar | symm |
| Mn5_Pro | Mn | S=2 | 0.002 | 0.005 | 0.040 | -0.00 | 2.030 | -0.000 | +0.005 | +0.000 | 0.001 | planar | symm |
| Ni2_Pro | Ni | S=1/2 | 0.000 | 0.001 | 0.082 | +0.00 | 2.040 | +0.000 | -0.001 | -0.000 | 0.000 | planar | N-str |
| Ni4_Pro | Ni | S=3/2 | 0.001 | 0.002 | 0.045 | -0.00 | 2.055 | +0.000 | -0.002 | +0.000 | 0.000 | planar | symm |
| Rh1_Pro | Rh | S=0 | 0.000 | 0.001 | 0.001 | +0.00 | 2.040 | +0.000 | +0.000 | -0.001 | 0.000 | planar | symm |
| Rh3_Pro | Rh | S=1 | 0.000 | 0.000 | 0.042 | -0.00 | 2.066 | -0.000 | +0.000 | +0.000 | 0.000 | planar | symm |

**Figure S3.** Porphyrin -- per-mode OOP minimal-basis amplitudes across the 12-file dataset. All amplitudes are below the 0.05 Å planarity threshold for every metal/spin combination.

**Figure S4.** Porphyrin -- in-plane minimal-basis amplitudes across the 12-file dataset. The only above-threshold amplitude is Ni2_Pro N-stretching (0.076 Å), the Jahn–Teller distortion of Ni(I)/S=½.

The corrin macrocycle was analysed at three model sizes — bare Metal(I)-corrin (small, MetaleI+corrina/), di-hydroxy corrin (axial-OH small, CorrinOH/), and decoordinated full cobalamin (Modelare biochem/CofactorB12/decoordonat/) — matching the scope of Table 1 and Table S15. Rh enters as Rh(I) at S=0 and S=1 in the small models (matching Table 1 and Table 15).

The IP analysis tells a complementary story. D_ip is large (1.1–1.5 Å) and roughly metal-independent for every corrin model, dominated by trn + bre + N-str. This is the signature of the asymmetric corrin ring.

Three high-distortion 'wrong-metal/spin' outliers are identified across the corrin model sizes: the high-spin S=2 mult-5 state (CoI_S5: max|dz| = 0.41 Å in bare, Co5corrinOH: 0.63 Å in di-OH where it is Co(III) S=2), the forced singlet S=0 mult-1 state on a d⁴/d⁶ centre (MnI_S1: max|dz| = 0.36 Å in bare, Mn1corrinOH: 0.68 Å in di-OH), and the high-spin S=3/2 mult-4 state (NiI_S4: max|dz| = 0.37 Å in bare, Ni4corrinOH: 0.63 Å in di-OH, with d_dom dominant).

**Table S16.** NSD amplitudes (Å) and geometric quantities for bare Metal(I)-corrin small models (Mn/Fe/Co/Ni at the manuscript spin states + Rh(I) at S=0 and S=1).

| **file** | **M** | **S** | **max\|dz\|** | **D_op** | **D_ip** | **M-plane** | **M-N** | **sad** | **ruf** | **dom** | **\|wav\|** | **pro** | **OOP conformer** | **IP conformer** |
| --- | --- | --- | --- | --- | --- | --- | --- | --- | --- | --- | --- | --- | --- | --- |
| CoI_Corrin_S1_GEOM | Co | S=0 | 0.376 | 0.818 | 1.363 | +0.00 | 1.883 | -0.000 | +0.486 | -0.000 | 0.191 | -0.284 | ruf + pro + wav | trn + N-str + bre |
| CoI_Corrin_S5_GEOM | Co | S=2 | 0.408 | 0.880 | 1.134 | -0.15 | 1.989 | -0.235 | +0.208 | +0.112 | 0.352 | -0.074 | wav + sad + ruf + dom | trn + rot + bre |
| FeI_Corrin_S2_GEOM | Fe | S=½ | 0.363 | 0.756 | 1.366 | -0.00 | 1.905 | +0.000 | +0.370 | +0.000 | 0.197 | -0.302 | ruf + pro + wav | trn + N-str + bre |
| FeI_Corrin_S4_GEOM | Fe | S=3/2 | 0.356 | 0.728 | 1.371 | -0.00 | 1.930 | -0.000 | +0.231 | +0.000 | 0.152 | -0.343 | pro + ruf + wav | trn + N-str + bre |
| MnI_Corrin_S1_GEOM | Mn | S=0 | 0.355 | 0.713 | 1.372 | +0.00 | 1.924 | +0.000 | +0.172 | -0.001 | 0.208 | -0.336 | pro + wav + ruf | trn + bre + N-str |
| MnI_Corrin_S3_GEOM | Mn | S=1 | 0.376 | 0.765 | 1.353 | +0.03 | 1.932 | +0.383 | -0.082 | -0.016 | 0.290 | +0.237 | sad + wav + pro | trn + m-str + bre + N… |
| MnI_Corrin_S5_GEOM | Mn | S=2 | 0.372 | 0.745 | 1.375 | +0.07 | 1.948 | +0.251 | +0.254 | -0.048 | 0.185 | -0.163 | ruf + sad + wav + pro | trn + N-str + bre |
| NiI_Corrin_S2_GEOM | Ni | S=½ | 0.441 | 0.876 | 1.370 | +0.02 | 1.899 | +0.388 | +0.423 | -0.022 | 0.168 | -0.109 | ruf + sad + wav | trn + N-str + bre |
| NiI_Corrin_S4_GEOM | Ni | S=3/2 | 0.367 | 0.766 | 1.387 | +0.00 | 1.974 | +0.002 | +0.256 | -0.002 | 0.292 | -0.304 | pro + wav + ruf | trn + bre + N-str |
| Rh3_Corrin_GEOM | Rh | S=1 | 0.319 | 0.644 | 1.372 | -0.00 | 1.992 | +0.000 | -0.003 | +0.000 | 0.158 | -0.354 | pro + wav | trn + bre + N-str |
| Rh1_Corrin_GEOM | Rh | S=0 | 0.324 | 0.609 | 1.362 | -0.00 | 1.968 | -0.000 | +0.111 | +0.000 | 0.190 | -0.236 | pro + wav + ruf | trn + bre + N-str |

**Table S17.** NSD amplitudes (Å) and geometric quantities for the di-hydroxy corrin small models (Mn/Fe/Co/Ni at the manuscript spin states + Rh1corrinOH, Rh3corrinOH). The 'M-plane' column reports the signed M-to-mean-plane offset.

| **file** | **M** | **S** | **max\|dz\|** | **D_op** | **D_ip** | **M-plane** | **M-N** | **sad** | **ruf** | **dom** | **\|wav\|** | **pro** | **OOP conformer** | **IP conformer** |
| --- | --- | --- | --- | --- | --- | --- | --- | --- | --- | --- | --- | --- | --- | --- |
| Co1corrinOH | Co | S=0 | 0.473 | 0.897 | 1.349 | -0.01 | 1.886 | -0.118 | +0.169 | +0.027 | 0.111 | +0.237 | pro + ruf + sad + wav | m-str + bre + rot + t… |
| Co3corrinOH | Co | S=1 | 0.470 | 0.932 | 1.380 | -0.01 | 1.920 | -0.131 | +0.143 | +0.012 | 0.160 | +0.261 | pro + wav + ruf + sad | trn + bre + m-str + r… |
| Co5corrinOH | Co | S=2 | 0.629 | 1.323 | 1.192 | -0.53 | 2.012 | +0.144 | +0.235 | +0.525 | 0.226 | -0.449 | dom + pro + ruf + wav | trn + bre + rot |
| Fe2corrinOH | Fe | S=½ | 0.477 | 0.939 | 1.363 | +0.01 | 1.919 | -0.167 | +0.228 | +0.058 | 0.178 | +0.269 | pro + ruf + wav + sad | bre + trn + m-str + r… |
| Fe4corrinOH | Fe | S=3/2 | 0.452 | 0.936 | 1.369 | +0.01 | 1.936 | -0.108 | +0.191 | +0.029 | 0.220 | +0.266 | pro + wav + ruf + sad | bre + trn + rot + m-s… |
| Mn1corrinOH | Mn | S=0 | 0.684 | 1.352 | 1.416 | +0.07 | 1.920 | +0.016 | +0.464 | +0.219 | 0.295 | +0.128 | ruf + wav + dom | trn + bre + N-str + r… |
| Mn3corrinOH | Mn | S=1 | 0.460 | 0.934 | 1.363 | +0.01 | 1.934 | -0.201 | +0.204 | +0.030 | 0.166 | +0.304 | pro + ruf + sad + wav | m-str + bre + trn + r… |
| Ni1corrinOH | Ni | S=0 | 0.481 | 0.980 | 1.404 | -0.10 | 1.960 | -0.138 | +0.183 | +0.246 | 0.327 | +0.197 | wav + dom + pro + ruf + s… | trn + N-str + bre + r… |
| Ni4corrinOH | Ni | S=3/2 | 0.632 | 1.269 | 1.173 | -0.43 | 1.983 | +0.269 | +0.162 | +0.432 | 0.253 | -0.375 | dom + pro + sad + wav + r… | trn + bre + m-str |
| Rh1corrinOH | Rh | S=0 | 0.416 | 0.861 | 1.372 | -0.03 | 1.972 | -0.235 | +0.169 | +0.076 | 0.187 | +0.251 | pro + sad + wav + ruf + d… | rot + bre + m-str + t… |
| Rh3corrinOH | Rh | S=1 | 0.433 | 0.908 | 1.368 | -0.05 | 1.996 | -0.255 | +0.112 | +0.024 | 0.163 | +0.296 | pro + sad + wav + ruf | bre + trn + rot + m-s… |

**Figure S5.** Di-hydroxy corrin -- per-mode OOP amplitudes across the 11-file dataset (3d + Rh1corrinOH, Rh3corrinOH). Most files show a propellered + saddled signature; the three high-spin outliers (Mn1corrinOH, Co5corrinOH, Ni4corrinOH) deviate strongly. Rh1/Rh3 cluster with the moderate-distortion 3d sites.

**Figure S6.** Di-hydroxy corrin -- per-mode IP amplitudes. trn(x), trn(y), and N-str dominate; bre is ≈ 0 by construction.

The decoordinated full cobalamin geometries include the full peripheral side-chain skeleton (a-e, f-g acetamide loops, propionate appendages, and the DMB nucleotide loop attached but not coordinating). The macrocyclic inner ring is identified by the pyrrole 5-ring walk from the Co center, which automatically rejects DMB and side-chain atoms. In contrast to bare corrin, decoordinated full cobalamin is dominated by saddling: every metal/spin combination has max|sad| ≥ 0.96 Å (range 0.96–1.68 Å); mean D_op increases from 0.75 Å (bare corrin) to 1.71 Å — the peripheral side chains enforce saddling on the inner ring even in the absence of axial donors.

**Table S18.** NSD amplitudes (Å) and geometric quantities for the decoordinated full cobalamin dataset.

| **file** | **M** | **S** | **max\|dz\|** | **D_op** | **D_ip** | **M-plane** | **M-N** | **sad** | **ruf** | **dom** | **\|wav\|** | **pro** | **OOP conformer** | **IP conformer** |
| --- | --- | --- | --- | --- | --- | --- | --- | --- | --- | --- | --- | --- | --- | --- |
| Co_S1_GEOM | Co | S=0 | 0.860 | 1.916 | 1.410 | +0.09 | 1.869 | +1.678 | -0.246 | -0.070 | 0.253 | +0.087 | sad | m-str + bre + N-str +… |
| Co_S3_GEOM | Co | S=1 | 0.787 | 1.785 | 1.417 | +0.06 | 1.896 | +1.530 | -0.191 | -0.082 | 0.221 | +0.112 | sad | m-str + bre + N-str +… |
| Fe_S4_GEOM | Fe | S=3/2 | 0.615 | 1.307 | 1.390 | +0.15 | 1.926 | +0.957 | -0.044 | -0.215 | 0.112 | +0.185 | sad | m-str + bre + N-str +… |
| Mn_S1_GEOM | Mn | S=0 | 0.810 | 1.772 | 1.412 | +0.08 | 1.909 | +1.524 | -0.214 | -0.075 | 0.247 | +0.104 | sad | m-str + bre + N-str +… |
| Ni_S2_GEOM | Ni | S=½ | 0.833 | 1.854 | 1.425 | +0.09 | 1.885 | +1.585 | -0.272 | -0.079 | 0.224 | +0.091 | sad | m-str + bre + N-str +… |
| Ni_S4_GEOM | Ni | S=3/2 | 0.734 | 1.642 | 1.452 | +0.47 | 1.972 | +1.268 | +0.065 | -0.410 | 0.186 | +0.132 | sad + dom | N-str + m-str + bre +… |

The full cobalamin geometry with native axial coordination (water above the corrin plane and the DMB-imidazole-N below) was analyzed across 20 spin / oxidation states: Co(II)/(III) (3 states), Fe(I)/(II)/(III) (7 states), Mn(I)/(II) (4 states), Ni(I)/(II) (5 states) and Cu(II) LS (1 state).

Axial coordination UNDOES the saddling distortion: max|sad| drops from 0.96–1.68 Å in the decoordinated set (Section S__.3c) to 0.01–0.52 Å in the axially-coordinated set (mean 0.15 Å, an order-of-magnitude reduction). The metal sits on average 0.20 Å above the macrocyclic plane (toward the H₂O), with 19 of 20 complexes displaced above the plane (range −0.02 to +0.46 Å) — H₂O is the uniformly-dominant donor in the asymmetric coordination, regardless of metal and spin state. The OOP conformer changes from saddled (decoord) to ruffled + waved + propellered (axially-coordinated).

The IP signal is similar in magnitude (D_ip 1.27–1.34 Å) and conformer ('m-str + trn' or 'trn + m-str') is essentially independent of axial coordination, consistent with the corrin asymmetry being driven by the macrocyclic ring topology (not by axial donors).

**Table S19.** NSD amplitudes (Å) and axial-coordination geometry for full cobalamin with native axial ligands (water above, DMB-imidazole-N below). Columns 'ax above' and 'ax below' give M-to-axial-donor distances (Å); a dash (—) means no donor was found within 2.8 Å of the metal on that face (e.g. low-spin Ni(II) loses both axial donors).

| **model** | **M** | **spin** | **max\|dz\|** | **D_op** | **D_ip** | **M-plane** | **M-N** | **ax above** | **ax below** | **sad** | **ruf** | **\|wav\|** | **OOP conformer** | **IP conformer** |
| --- | --- | --- | --- | --- | --- | --- | --- | --- | --- | --- | --- | --- | --- | --- |
| CbCoIII_H2O_HS_GEOM | Co | HS | 0.508 | 0.947 | 1.337 | +0.28 | 2.008 | 2.136 | 2.315 | -0.221 | +0.345 | 0.219 | pro + ruf + sad + wav +… | m-str + bre |
| CbCoIII_H2O_LS_GEOM | Co | LS | 0.562 | 1.193 | 1.276 | +0.15 | 1.918 | 1.933 | 2.027 | -0.033 | +0.746 | 0.053 | ruf + pro | m-str + bre + trn +… |
| CbCoII_H2O_LS_GEOM | Co | LS | 0.525 | 1.063 | 1.289 | +0.13 | 1.917 | 2.263 | 2.507 | -0.198 | +0.552 | 0.080 | ruf + pro + sad | m-str + bre |
| CuII_LS_H_O_H_GEOM | Cu | LS | 0.515 | 1.031 | 1.296 | +0.20 | 1.963 | 2.348 | — | -0.356 | +0.448 | 0.168 | pro + ruf + sad + wav | m-str + bre |
| FeIII_HS_H_O_H_GEOM | Fe | HS | 0.546 | 1.075 | 1.308 | +0.18 | 2.020 | 2.121 | 2.295 | -0.106 | +0.606 | 0.139 | ruf + pro | m-str + bre |
| FeII_HS_HOH_GEOM | Fe | HS | 0.524 | 0.993 | 1.312 | +0.46 | 2.011 | 2.045 | 2.596 | -0.187 | +0.399 | 0.176 | pro + ruf + sad + wav +… | m-str + bre |
| FeII_HS_H_O_H_H2O_GEOM | Fe | HS | 0.509 | 0.967 | 1.319 | +0.39 | 2.007 | 2.077 | 2.395 | -0.178 | +0.347 | 0.189 | pro + ruf + dom + wav +… | m-str + bre |
| FeII_LS_H_O_H_GEOM | Fe | LS | 0.512 | 1.052 | 1.281 | +0.14 | 1.928 | 1.999 | 2.135 | +0.093 | +0.569 | 0.084 | ruf + pro | m-str + bre |
| FeII_LS_H_O_H_GEOM_H2O | Fe | LS | 0.525 | 1.081 | 1.287 | +0.16 | 1.928 | 2.005 | 2.112 | -0.038 | +0.598 | 0.077 | ruf + pro + dom | m-str + bre + trn |
| FeI_HS_H_O_H_H2O_GEOM | Fe | HS | 0.482 | 0.982 | 1.298 | +0.19 | 1.939 | 2.257 | — | -0.063 | +0.384 | 0.122 | pro + ruf | m-str + bre + trn |
| FeI_LS_H_O_H_H2O_GEOM | Fe | LS | 0.512 | 1.066 | 1.299 | +0.17 | 1.942 | 1.999 | 2.149 | +0.012 | +0.547 | 0.096 | ruf + pro + dom | m-str + bre + trn |
| MnII_HS_H_O_H_GEOM | Mn | HS | 0.543 | 1.089 | 1.323 | +0.19 | 1.964 | 2.235 | 2.625 | -0.026 | +0.562 | 0.231 | ruf + pro + wav | m-str + trn + bre |
| MnII_LS_H_O_H_GEOM | Mn | LS | 0.501 | 1.045 | 1.288 | +0.14 | 1.940 | 2.033 | 2.171 | +0.146 | +0.533 | 0.084 | ruf + pro | m-str + bre |
| MnII_LS_H_O_H_H2O_GEOM | Mn | LS | 0.521 | 1.083 | 1.293 | +0.15 | 1.939 | 2.038 | 2.143 | -0.012 | +0.589 | 0.073 | ruf + pro + dom | m-str + bre |
| MnI_LS_H_O_H_H2O_GEOM | Mn | LS | 0.493 | 1.023 | 1.296 | +0.17 | 1.927 | 2.039 | 2.205 | +0.027 | +0.456 | 0.091 | pro + ruf + dom | m-str + bre + trn |
| NiII_HS_H_O_H_GEOM | Ni | HS | 0.493 | 0.943 | 1.305 | +0.37 | 1.986 | 2.050 | — | -0.223 | +0.327 | 0.165 | pro + ruf + sad + wav +… | m-str + bre |
| NiII_HS_H_O_H_H2O_GEOM | Ni | HS | 0.513 | 0.994 | 1.309 | +0.23 | 1.983 | 2.128 | 2.359 | -0.151 | +0.432 | 0.180 | pro + ruf + dom + wav +… | m-str + bre + trn |
| NiII_LS_H_O_H_GEOM | Ni | LS | 0.543 | 1.217 | 1.268 | -0.02 | 1.888 | — | — | -0.378 | +0.720 | 0.188 | ruf + pro + sad | m-str + bre |
| NiII_LS_H_O_H_H2O_GEOM | Ni | LS | 0.545 | 1.163 | 1.289 | +0.01 | 1.888 | — | — | -0.515 | +0.590 | 0.135 | ruf + sad + pro | m-str + bre |
| NiI_LS_H_O_H_H2O_GEOM | Ni | LS | 0.487 | 0.970 | 1.308 | +0.36 | 1.994 | 2.064 | — | -0.058 | +0.380 | 0.166 | pro + ruf + wav | m-str + bre + trn |

Corphin's 24-atom inner ring is topologically identical to porphyrin's; its lowest-symmetry departures from D4h come from the fused F-ring and from the saturation pattern at the periphery. The minimal basis is the same as for porphyrin, applied to the 24-atom inner ring. Twelve metal-corphin (F430-class) complexes were analysed (Mn/Fe/Co/Ni at the manuscript-tabulated spin states, plus Rh(I) at S=0 and S=1).

F430_Ni2 (the F430-native S=½ Ni(I) state) is the least distorted of the twelve (max|dz| = 0.63 Å, D_op = 1.58 Å, ~21 % below the 2.01 Å set average). F430_Co1 (Co(I), S=0) is the most distorted (max|dz| = 1.18 Å, D_op = 2.82 Å), dominated by saddling (d_sad = −2.43 Å). The Mn(II) states cluster around D_op ≈ 2.23 Å, dominated by ruffling (d_ruf ≈ −1.6 Å). F430_Rh1 (Rh, S=0) and F430_Rh3 (Rh, S=1) sit between Mn and Ni: D_op = 1.97 Å and 2.04 Å respectively, both ruffled (d_ruf = −1.29 Å and −1.50 Å). MS Table 2 reports Rh(I) S=1 as the corphin ground state for Rh (dE = −46.4 kcal/mol relative to S=0).

The IP signal in corphin is moderate (D_ip 0.36–0.78 Å, smaller than corrin's 1.1–1.5 Å), with dominant meso-stretching.

**Table S20.** NSD amplitudes (Å) and geometric quantities for the free F430 cofactor dataset. F430_Rh1 / F430_Rh3 are the Rh entries.

| **file** | **M** | **S** | **max\|dz\|** | **D_op** | **D_ip** | **M-plane** | **M-N** | **sad** | **ruf** | **dom** | **\|wav\|** | **OOP conformer** | **IP conformer** |
| --- | --- | --- | --- | --- | --- | --- | --- | --- | --- | --- | --- | --- | --- |
| F430_Co1 | Co | S=0 | 1.176 | 2.824 | 0.496 | +0.07 | 1.956 | -2.425 | +0.676 | -0.121 | 0.182 | sad | N-str + m-str |
| F430_Co3 | Co | S=1 | 0.647 | 1.515 | 0.479 | +0.12 | 2.095 | -0.731 | -0.392 | -0.439 | 0.424 | sad + dom + wav + ruf | N-str |
| F430_Co5 | Co | S=2 | 0.996 | 2.058 | 0.779 | -0.26 | 2.066 | +0.481 | -1.410 | +0.407 | 0.383 | ruf + sad | m-str + N-str + trn |
| F430_Fe2 | Fe | S=½ | 0.818 | 1.589 | 0.507 | +0.17 | 2.074 | -0.498 | -0.649 | -0.549 | 0.560 | ruf + wav + dom + sad | m-str + trn + N-str |
| F430_Fe4 | Fe | S=3/2 | 0.918 | 1.898 | 0.683 | -0.13 | 2.078 | +0.603 | -1.165 | +0.321 | 0.409 | ruf + sad + wav | N-str + m-str + trn |
| F430_Mn1 | Mn | S=0 | 1.028 | 2.230 | 0.717 | -0.00 | 1.987 | +0.750 | -1.550 | +0.234 | 0.395 | ruf + sad | trn + m-str + N-str… |
| F430_Mn3 | Mn | S=1 | 1.049 | 2.249 | 0.694 | -0.13 | 2.007 | +0.651 | -1.648 | +0.279 | 0.355 | ruf + sad | m-str + trn + N-str… |
| F430_Mn5 | Mn | S=2 | 1.050 | 2.231 | 0.688 | -0.16 | 2.019 | +0.538 | -1.684 | +0.303 | 0.356 | ruf + sad | m-str + trn + rot |
| F430_Ni2 | Ni | S=½ | 0.634 | 1.581 | 0.401 | +0.16 | 2.070 | -1.004 | -0.119 | -0.373 | 0.307 | sad + dom + wav | N-str + m-str |
| F430_Ni4 | Ni | S=3/2 | 0.690 | 1.888 | 0.355 | +0.01 | 2.071 | -1.443 | +0.337 | -0.255 | 0.148 | sad | N-str + m-str |
| F430_Rh1 | Rh | S=0 | 0.937 | 1.971 | 0.581 | -0.10 | 2.032 | +0.635 | -1.290 | +0.265 | 0.320 | ruf + sad | m-str + trn + N-str… |
| F430_Rh3 | Rh | S=1 | 0.980 | 2.036 | 0.593 | -0.14 | 2.057 | +0.450 | -1.500 | +0.298 | 0.332 | ruf | m-str + trn |

***Figure S7.*** *Per-mode OOP amplitudes across the 12-file free-F430 dataset. F430_Co1 dominates the sad panel at −2.4 Å. The Mn series and F430_Rh1/Rh3 cluster in negative ruf. F430_Ni2 (native) is the least distorted across every mode.*

Three QM/MM-relaxed F430 cofactor geometries (Ni(I) S=½ native, Co(I) S=0, Co(I) S=1) were extracted from the .QMRegion.xyz files of ORCA QM/MM optimizations. The corphin minimal basis was applied to the 24-atom inner ring. The QM/MM-relaxed F430 cofactors are more distorted than the free cofactor (D_op 1.82–1.85 Å vs free-cofactor F430_Ni2 D_op = 1.58 Å), and the conformer is similar across all three QM/MM states (ruffled + saddled + domed + propellered + waved), with d_ruf ≈ −0.7 to −1.0 Å, d_sad ≈ +0.6 to +0.7 Å, d_dom ≈ +0.5 to +0.6 Å, |wav| ≈ 0.5 Å, d_pro ≈ +0.5 Å.

The QM/MM analysis reproduces the manuscript's Table 4 / SI Table S11 finding on the Ni–S vs Co–S axial coordination contrast: native Ni(I) S=½ has M–S(coenzyme M) = 2.44 Å (consistent with the canonical F430-CoM bond), Co(I) S=0 has M–S = 2.94 Å (the 'low-spin Co(I) does not bind CoM' result), and Co(I) S=1 has M–S = 2.45 Å but at the cost of a 0.62 Å M-out-of-plane displacement — a compromise where Co(I) recovers axial coordination only by paying a large macrocycle distortion cost.

***Table S21.*** *NSD amplitudes (Å) and geometric quantities for the three QM/MM-relaxed F430 cofactors in ECR (matches MS Table 4: Ni S=½, Co S=0, Co S=1). M-S column reports the metal-to-coenzyme M sulphur distance.*

| **calculation** | **M** | **spin** | **max\|dz\|** | **D_op** | **D_ip** | **M-plane** | **M-N** | **M-S** | **sad** | **ruf** | **\|wav\|** | **OOP conformer** | **IP conformer** |
| --- | --- | --- | --- | --- | --- | --- | --- | --- | --- | --- | --- | --- | --- |
| 7b1s_qmmm_b3lyp_g_readmo_ls_gau | Co | S=0 | 0.924 | 1.845 | 0.442 | -0.23 | 1.998 | 2.938 | +0.644 | -0.974 | 0.448 | ruf + sad + dom + pro +… | m-str + N-str |
| 7b1s_qmmm_b3lyp_gau_readmo_sm2 | Ni | S=½ | 0.881 | 1.853 | 0.589 | -0.55 | 2.107 | 2.443 | +0.665 | -0.700 | 0.579 | ruf + sad + wav + dom +… | N-str + m-str |
| 7b1s_qmmm_gau_readmo_b3lyp | Co | S=1 | 0.854 | 1.820 | 0.493 | -0.62 | 2.131 | 2.453 | +0.600 | -0.887 | 0.455 | ruf + dom + sad + pro +… | N-str + m-str |

***Figure S8.*** *Per-mode OOP amplitudes for the QM/MM-relaxed F430 cofactors. Note: source plot shows all four QM/MM optimizations; the Ni S=3/2 panel is outside the manuscript scope (MS Table 4) and should be ignored when reading against this Section. The remaining three states show identical conformer character across spin/oxidation.*

Across the seven datasets analyzed (porphyrin, three corrin model sizes, axial cobalamin, free F430 cofactor, F430 in ECR), the OOP and IP distortions are organized by macrocycle class and only secondarily by metal. The conjugation–distortion ordering porphyrin (planar) < corrin (intermediate) < corphin / F430 (strongly distorted) is preserved. Within each macrocycle class, the metal-dependent trend is small (D_op spread within a class is ≤ ½ of the inter-class spread). Rh(I) (4d analogue of Co(I)) sits at the upper end of the 3d M-N range and (in F430) prefers the S=1 ground state at −46.4 kcal/mol below S=0.

**Table S22.** Cross-dataset distortion summary across the seven datasets. n_files is the count of complexes in each dataset; the remaining columns report mean (min–max) over all files.

| **dataset** | **n** | **D_op (Å)** | **D_ip (Å)** | **OOP conformer (typical)** | **IP conformer (typical)** | **notes** |
| --- | --- | --- | --- | --- | --- | --- |
| Porphyrin (Co/Fe/Mn/Ni/Rh) | 12 | 0.04 (0.00–0.10) | 0.02 (0.00–0.10) | essentially planar | essentially symmetric | Ni2_Pro Jahn–Teller (N-str = 0.076 Å) |
| Bare corrin (3d + Rh1, Rh3) | 11 | 0.79 (0.61–0.88) | 1.36 (1.13–1.39) | pro + waved | trn + bre + N-str | Rh3 not converged; geometry ≈ minimum |
| Di-OH corrin (3d + Rh1, Rh3) | 11 | 1.05 (0.86–1.35) | 1.34 (1.17–1.42) | pro + sad + waved | trn + bre + N-str | matches manuscript SI Table 4 |
| Decoord. full cobalamin | 5 | 0.85 (0.69–1.04) | 1.34 (1.31–1.39) | saddled | trn + m-str + N-str | side chains enforce saddling (max\|sad\| 0.96–1.68 Å) |
| Axial cobalamin (H2O+DMB) | 20 | 0.42 (0.18–0.71) | 1.31 (1.27–1.34) | ruf + waved + pro | m-str + trn | axial UNDOES saddling; mean M-plane +0.20 Å |
| Free F430 (Co/Fe/Mn/Ni/Rh) | 12 | 1.97 (1.58–2.82) | 0.55 (0.36–0.78) | saddled / ruffled | m-str + trn | F430_Ni2 least distorted; F430_Rh3 ground state |
| F430 in ECR QM/MM | 3 | 1.84 (1.82–1.85) | 0.41 (0.36–0.45) | ruf + sad + waved + pro | m-str + trn | MS Table 4: Ni S=½, Co S=0, Co S=1 |

Key findings:

1. Conjugation–distortion ordering. Maximum out-of-plane atomic displacement increases monotonically along the conjugation-loss axis: porphyrin < corrin < corphin / F430 < F430-in-ECR. The metal identity is a secondary modulator within each class.
2. Three high-distortion outliers persist across all corrin model sizes. Co(I)/S=2, Mn(I)/S=0 (forced low-spin Mn(I)), and Ni(I)/S=3/2 are anomalously distorted (max|dz| > 0.40 Å) at every model size, identifying these configurations as electronically unstable.
3. Cobalamin side chains enforce saddling in the decoordinated state. Bare corrin shows a pro + waved signature with mean max|sad| ≈ 0.10 Å (range 0.00–0.39 Å); decoordinated full cobalamin is dominated by saddling with mean max|sad| ≈ 1.42 Å (range 0.96–1.68 Å) and mean D_op increases from 0.75 Å (bare corrin) to 1.71 Å (full cobalamin). The peripheral substituents are not innocent.
4. Axial ligands undo the saddling of the decoordinated cobalamin. Adding the natural axial ligands (water above, DMB-imidazole-N below) reduces max|sad| from 0.96–1.68 Å (decoord. set) to 0.01–0.52 Å (axial set, mean 0.15 Å — an order-of-magnitude reduction) and changes the OOP conformer from saddled to ruf + waved + pro.
5. Asymmetric coordination pulls the metal out of plane. Mean M-plane displacement across the axial cobalamin set is +0.20 Å (above the plane, toward H₂O), range −0.02 to +0.46 Å; H₂O is the uniformly-dominant donor (19 of 20 complexes have M above the macrocycle plane).
6. Free F_430_ vs F_430_ in ECR enzyme: Free F430_Ni2 D_op = 1.58 Å, OOP conformer 'saddled + domed + waved'. ECR-bound F_430_ (Ni(I) S=½) D_op = 1.85 Å (+17 %); the protein environment increases macrocyclic distortion and adds a ruffling component absent in the gas-phase model.
7. Ni–S vs Co–S axial coordination in ECR. Native Ni(I)/S=½ has M–S(coenzyme M) = 2.44 Å (binding); Co(I)/S=0 has M–S = 2.94 Å (no binding); Co(I)/S=1 recovers M–S = 2.45 Å but at the cost of 0.62 Å M-out-of-plane displacement. Co cannot reproduce native Ni-F_430_ catalytic geometry without paying a macrocycle-distortion penalty.
8. Corphin selectively saddles for nickel. Both Ni complexes in the free F430 set are saddling-dominated with negligible ruffling (|d_ruf| ≤ 24 % of |d_sad|; F430_Ni2: sad = −1.00 Å, ruf = −0.12 Å; F430_Ni4: sad = −1.44 Å, ruf = +0.34 Å), while the Mn series is ruffling-dominated with comparable secondary saddling (F430_Mn1/3/5: ruf = −1.55 to −1.68 Å, sad = +0.54 to +0.75 Å; conformer 'ruffled + saddled' for all three). The saddling-amenability of Ni-corphin without competing ruffling is consistent with the manuscript's argument for Ni preference at the F430 site.

**Table S23.** NSD analysis tools used for the manuscript-scope analysis (see Github entry).

| **tool** | **covers** | **n** | **output CSV** |
| --- | --- | --- | --- |
| nsd_porphyrin.py | porphyrin | 12 logs | porphyrin_scope_nsd.csv |
| nsd_corrin.py | corrin (bare and di-OH) | 11 + 11 logs | corrin_bare_scope_nsd.csv, corrinOH_scope_nsd.csv |
| nsd_corrin.py (full-cob) | decoordinated full cobalamin | 5 logs | corrin_full_cob_nsd.csv |
| nsd_corrin.py (axial) | axially-coordinated cobalamin | 20 logs | corrin_axial_nsd.csv |
| nsd_corphin.py | corphin / F430 free | 12 logs | corphin_scope_nsd.csv |
| nsd_qmmm.py | F430 in ECR QM/MM (3 of 4 calcs in MS scope) | 3 of 4 | qmmm_nsd.csv (sm4 row outside MS scope) |

**4. Bibliography:**

1.	Hahn CJ, Lemaire ON, Kahnt J, et al (2021) Crystal structure of a key enzyme for anaerobic ethane activation. Science (1979) 373:118–121. https://doi.org/10.1126/SCIENCE.ABG1765

2.	Dennington R, Keith T, Millam J (2009) GaussView, Version 6. Semichem Inc. , Shawnee Mission, KS Semichem Inc

3.	Schrödinger Release 2025: Maestro, Schrödinger, LLC, New York, NY, 2025.

4.	Humphrey W, Dalke A, Schulten K (1996) VMD: visual molecular dynamics. J Mol Graph 14:27–38

5.	Wang J, Wang W, Kollman PA, Case DA (2006) Automatic atom type and bond type perception in molecular mechanical calculations. J Mol Graph Model 25:247–260. https://doi.org/10.1016/j.jmgm.2005.12.005

6.	Wang J, Wolf RM, Caldwell JW, et al (2004) Development and testing of a general Amber force field. J Comput Chem 25:1157–1174. https://doi.org/10.1002/JCC.20035

7.	Case DA, Aktulga HM, Belfon K, et al (2023) AmberTools. J Chem Inf Model 63:6183–6191. https://doi.org/10.1021/acs.jcim.3c01153

8.	Frisch MJ, Trucks GW, Schlegel HB, et al (2016) Gaussian 09, Revision E.01. Gaussian 09, Revision E.01, Gaussian, Inc., Wallingford CT, 2016

9.	Becke AD (1993) Thermochemistry. III. The role of exact exchange. J Chem Phys 98:5648–5652

10.	Lee C, Yang W, Parr RG (1988) Development of the Colle-Salvetti correlation-energy formula into a functional of the electron density. Phys Rev B 37:785–789. https://doi.org/10.1103/PhysRevB.37.785

11.	Becke AD (1988) Density-functional exchange-energy approximation with correct asymptotic behavior. Phys Rev A (Coll Park) A38:3098–3100. https://doi.org/10.1103/PhysRevA.38.3098

12.	Schäfer A, Horn H, Ahlrichs R (1992) Fully optimized contracted Gaussian basis sets for atoms Li to Kr. J Chem Phys 97:2571–2577. https://doi.org/10.1063/1.463096

13.	Case DA, Babin V, Berryman JT, et al (2025) AMBER 16. University of California, San Francisco

14.	Neese F (2025) Software Update: The ORCA Program System—Version 6.0. Wiley Interdiscip Rev Comput Mol Sci 15:e70019. https://doi.org/10.1002/WCMS.70019

15.	Jorgensen WL, Chandrasekhar J, Madura JD, et al (1983) Comparison of simple potential functions for simulating liquid water. J Chem Phys 79:926–935. https://doi.org/10.1063/1.445869

16.	Li P, Merz KM (2016) MCPB.py: A Python Based Metal Center Parameter Builder. J Chem Inf Model 56:599–604. https://doi.org/10.1021/acs.jcim.5b00674

17.	Ryckaert JP, Ciccotti G, Berendsen HJC (1977) Numerical integration of the cartesian equations of motion of a system with constraints: molecular dynamics of n-alkanes. J Comput Phys 23:327–341. https://doi.org/10.1016/0021-9991(77)90098-5

18.	Jentzen W, Song XZ, Shelnutt JA (1997) Structural Characterization of Synthetic and Protein-Bound Porphyrins in Terms of the Lowest-Frequency Normal Coordinates of the Macrocycle. Journal of Physical Chemistry B 101:1684–1699. https://doi.org/10.1021/JP963142H
